# Editorial: Women in nanotoxicology 2023–2024

**DOI:** 10.3389/ftox.2025.1599767

**Published:** 2025-05-06

**Authors:** Iseult Lynch, Ilika Ghosh

**Affiliations:** ^1^ School of Geography, Earth and Environmental Sciences, University of Birmingham, Birmingham, United Kingdom; ^2^ Neuroenergetics Group, Max Planck Florida Institute for Neuroscience, Jupiter, FL, United States

**Keywords:** nanotoxicology, nanosafety, women in STEM, fate and behavior, adverse outcome pathway (AOP)

Over the past few decades, there has been a significant effort made to recognize and highlight the invaluable contributions of women in STEM (Science, Technology, Engineering, and Mathematics) fields. Historically, women have been underrepresented in these areas due to systemic barriers, cultural biases, and limited access to opportunities. Indeed, recent analysis of the impact of the naming of scientific prizes after males, neutral names or after females on the proportion of female awardees was illuminating: Only 11.8% of prizes and awards were awarded to woman scientists if the respective prizes or medals were named after a man (consistent across 345 scientific medals and prizes awarded by 11 General Scientific Societies) whereas for awards that did not bear the name of a specific individual or were named after a woman, the proportion of woman recipients was considerably higher (31.8% and 46.9%, respectively) (Gehmlich and Krause, 2024a; Gehmlich and Krause, 2024b). Similarly, until very recently, less than 20% of Wikipedia biographies were about women (Wade and Zaringhalam, 2018). As 11 February 2025, marked the International Day of Women and Girls in Science, women still represent only one-third of the global research community according to UNESCO (Azoulay, 2025). A large-scale analysis of author lists of scientific papers found that while women make up nearly 50% of the workforce the likelihood of a woman being credited on a paper is 13% lower than the men and the effort and scale of the input needed to gain authorship is much higher (Ross et al., 2022).

However, the landscape is slowly changing as more women are breaking through these barriers, leading groundbreaking research, and assuming leadership roles in academia, industry, and policy-making. In nanotoxicology, women have been at the forefront of pioneering studies that explore the effects of nanomaterials on human and environmental health. Indeed, the first paper suggesting that nanoscale materials might behave differently in the environment than dissolved chemicals or larger particles was led by Dr. Vicki Colvin then at Rice University (Colvin, 2003). Many of the early pioneering nanomaterials toxicity studies were led by women (Lovern and Klaper, 2006; Wang et al., 2008; Gaiser et al., 2009; Shvedova et al., 2005), who also recognized the wider implications including the potential for disproportionate impacts of nanotechnology on vulnerable populations, advocating for equitable and sustainable practices in the field (Invernizzi and Foladori, 2005; Dalton-Brown, 2012). Their work has not only advanced our understanding of mechanisms of toxicity of nanomaterials but has also contributed to the development of safer nanomaterials, improved risk assessment methodologies, and innovative therapeutic strategies. This growing recognition is a testament to the resilience, creativity, and dedication of women in science.

The four articles included in the Research Topic span the breadth of the nanotoxicology domain from chemistry and materials science to environmental science and biology, reflecting the inherent interdisciplinary of nanotoxicology. The Research Topic involves 31 individual authors, 77% of whom are women, with all four of the senior authors being women and three of the first authors also being women. The papers span from green synthesis of silver and iron composite nanomaterials (Bashir et al.), to evaluation of the impact of phytoplankton secretions on the fate of engineered nanomaterials (Gasco and Slaveykova), to assessing the impact of oral exposure to solid lipid nanoparticle drug carriers on reproductive success in mice (Lacconi et al.) and leveraging omics data to strengthen understanding of chemical-biological interactions in different biological systems (del Guidice et al.). A summary of the main findings from each paper, and their broader implications, is given below.

As part of the push to produce nanomaterials for environmental remediation from greener raw materials and using low energy production routes, Bashir et al. highlight the use of an aqueous extract of *Zanthoxylum Armatum s*eeds (also called winged prickly ash or rattan pepper) which acts as a reducing, stabilizing, and capping agent for particle synthesis. The resulting nanocomposite of silver and Iron were evaluated for their efficacy in removal of Acid Black 234 dye, which is widely used in textile and leather dying, from wastewater. The results demonstrated 98% dye removal from the wastewater sample within 60 min, highlighting that the potential of the nanocomposites as an efficient and cost-effective solution for mitigating environmental pollution.

An exploration of the role of phytoplankton sections on nanomaterials fate in the aquatic environment by Gasco and Slaveykova highlighted a major knowledge gap in current knowledge in particular in terms of the regulatory mechanism and exometabolite changes due to the exposure of phytoplankton species to metal-based nanomaterials. Given that the chemical conditions and nanomaterials stability will be strongly affected by phytoplankton secretions in the microenvironment surrounding phytoplankton cells in comparison with bulk waters, unravelling the significance of secreted biomolecules in modulating the behaviour of the metal-containing nanomaterials is central for understudying the phytoplankton-nanomaterials feedback loops, drivers of nanomaterials transformations and their mechanisms of toxicity in the aquatic environment.

Ensuring that nanoparticle-based drug delivery systems are safe for use in women of reproductive age, and don’t compromise reproductive health is essential. Lacconi et al. evaluated whether repeated oral administration of solid lipid nanoparticles to female mice prior to mating would influence key pregnancy outcomes using mice as a model. CD1 female mice were exposed at two different dosages—low (7.5 mg/kg) and high (750 mg/kg) —three times a week for 6 weeks, following which female mice were mated and pregnancy was monitored from conception to delivery. The results showing that both loaded (with the target therapeutic load) and unloaded solid lipid nanoparticles did not affect the integrity of the simulated intestinal epithelial barrier, and that administering solid lipid nanoparticles as a drug delivery vehicle, prior to conception does not affect either maternal health or foetal development, posing no risk to future pregnancy.

As part of the drive to enhance mechanistic understanding of the impacts of nanomaterials and to facilitate effects-based grouping and read-across, including through use of the Adverse Outcome Pathway (AOP) framework, omics technologies are emerging as a critical tool to support more accurate risk assessments. The perspective paper of del Guidice et al. provides a roadmap towards integration of omics data into regulatory risk assessment as a means to strengthen understanding of the responses of different biological systems, emphasizing holistic chemical-biological interactions. The authors call for meticulous test system characterization: when developing *in vitro* methods interpretation of the mechanism of action of chemical exposures should be contextualized with the biological system used to generate the molecular profile, as this would allow a better understanding of the (partial) effect of the substance and provide a better prediction of phenotype variability and contribution to multiple adverse outcomes. To stably implement the use of omics data derived information, the robustness and generalizability of *in vitro* assays and omics profiles must be ensured, and the authors suggest that implementation of Good Laboratory Practice (GLP) principles for omics data generation would boost transparency, reproducibility, and reliability while ensuring standardization of experimental planning.

This Research Topic, the first of many such biannual Research Topic for materials toxicity and safety assessment, is not only a celebration of their work but also a call to action to continue to support and empower women in STEM for generations to come. The growing recognition of women’s contributions in nanotoxicology is a positive step toward a more equitable and innovative scientific community, but there is still much work to be done to overcome the biases noted above, and many others. By celebrating women’s achievements and addressing the barriers they face, journals can play a pivotal role in advancing both the science and the achievement of an equitable representation and voice.

We are extremely grateful to the authors that submitted their research to the Research Topic, to the reviewers who provided insightful and constructive feedback and suggestions for improvement, and to the editorial team for their contributions to making the Research Topic happen. We hope that you, the readers, will find the topic inspiring and will continue the challenge of pushing the boundaries of knowledge in nanotoxicology, ensuring that the collective knowledge feeds forward into the fields of advanced materials and microplastics, and in pushing the visibility and recognition of women in nanotoxicology and beyond, and the critical role of women in shaping the future of nanotoxicology.
